# Ramucirumab, another anti-angiogenic agent for metastatic colorectal cancer in second-line setting—its impact on clinical practice

**DOI:** 10.1186/s13045-015-0183-8

**Published:** 2015-07-28

**Authors:** Gaurav Goel, Weijing Sun

**Affiliations:** Division of Hematology-Oncology, Department of Medicine, University of Pittsburgh Cancer Institute, University of Pittsburgh School of Medicine, 5150 Centre Avenue, Fifth Floor, Pittsburgh, PA 15232 USA

**Keywords:** Anti-angiogenesis, Ramucirumab, Bevacizumab, Ziv-aflibercept, Colorectal cancer

## Abstract

The recent FDA approval of ramucirumab (RAISE trial) has added a third agent to our existing armamentarium of angiogenesis inhibitors (bevacizumab and ziv-aflibercept) for the second-line treatment of metastatic colorectal cancer, which may have some impacts in the current clinic practice.

The recent FDA approval of ramucirumab (a fully human IgG1 monoclonal antibody against the vascular endothelial growth factor receptor [VEGFR]-2 extracellular domain), in combination with FOLFIRI (RAISE trial) [1], has added a third agent to our armamentarium of angiogenesis inhibitors (bevacizumab and ziv-aflibercept) in the second-line treatment of metastatic colorectal cancer (mCRC). This has also resulted in further crowding of the existing treatment landscape, especially in the current scenario where these anti-VEGF agents are characterized by comparable efficacy and similar toxicity profiles.

Angiogenesis plays a crucial role in colorectal tumorigenesis, and the VEGF pathway is the only target that has been validated to date [2]. The VEGF family of ligands consists of a number of glycoproteins including VEGF-A, VEGF-B, VEGF-C, VEGF-D, and placental growth factor (PlGF). They bind to their cognate receptors VEGFR-1, VEGFR-2, and VEGFR-3 leading to intracellular signaling with resultant endothelial proliferation and migration. Before the approval of ramucirumab, several targeted agents aimed at inhibiting VEGF signaling have been developed for the treatment of mCRC, including antibody-mediated inhibition of ligand binding to the target VEGF receptors (bevacizumab; IgG1 Fc-VEGF receptor construct, ziv-aflibercept) and inhibitor of intracellular receptor tyrosine kinases of VEGFRs (regorafenib) [3, 4]. A series of phase III clinical trials have confirmed the efficacy of these VEGF inhibition strategies in the treatment of mCRC. Consequently, the use of anti-angiogenic treatments in conjunction with chemotherapy has become an accepted standard of care in mCRC.

ML18147 (a study of Avastin [bevacizumab] plus crossover fluoropyrimidine-based chemotherapy in patients with metastatic colorectal cancer) was the first study to demonstrate the benefit of continuing anti-angiogenic agent bevacizumab (in combination with chemotherapy) as a second-line therapy, even after previous exposure to the agent [5]. The VELOUR trial (aflibercept versus placebo in combination with irinotecan and 5-FU in the treatment of patients with metastatic colorectal cancer after failure of an oxaliplatin-based regimen) established the efficacy of ziv-aflibercept and FOLFIRI combination in mCRC patients who had progressed on oxaliplatin-containing chemotherapy [6]. The recent phase III RAISE study (ramucirumab versus placebo in combination with second-line FOLFIRI in patients with metastatic colorectal carcinoma that progressed during or after first-line therapy with bevacizumab, oxaliplatin, and a fluoropyrimidine) demonstrated that ramucirumab in combination with FOLFIRI significantly prolonged overall survival (OS; 13.3 vs. 11.7 months, hazard ratio [HR] = 0.84, 95 % confidence interval [CI] 0.73–0.98, *P* = 0.0219) and progression-free survival (PFS; 5.7 vs. 4.5 months, HR = 0.79, 95 % CI 0.70–0.90, *P* < 0.0005) in patients with mCRC whose disease had progressed during or after first-line treatment with bevacizumab, oxaliplatin, and a fluoropyrimidine [1].

A careful review of results indicates noteworthy similarities between these three studies. All of these trials demonstrated a benefit of combining an anti-VEGF agent (bevacizumab, ziv-aflibercept, or ramucirumab) with chemotherapy beyond initial progression in patients with mCRC. Although cross-trial comparison suffers from inherent limitations and should be interpreted with caution, it is interesting to note that the three anti-VEGF agents evaluated in these trials exhibited a similar improvement in OS (TML 1.4 months, VELOUR 1.4 months, RAISE 1.6 months) and PFS (TML 1.7 months, VELOUR 2.2 months, RAISE 1.2 months). The stratified HR for OS were also quite similar in the TML (0.83), VELOUR (0.82), and RAISE (0.84) trials. Moreover, the toxicity profiles of these agents overlapped, with a higher incidence of anti-VEGF-associated adverse events (such as hemorrhage, hypertension, and proteinuria) in the anti-angiogenesis agent arms, as was expected.

There were, however, a few important dissimilarities noted as well, which were primarily related to the treatment regimens used in these studies. In the VELOUR and RAISE trials, all patients received oxaliplatin- and fluoropyrimidine-based regimens as first-line treatment. In the ML18147 study, approximately 60 % of patients received irinotecan-based, and the remaining 40 % received oxaliplatin-based regimen as the first-line therapy. All patients in the ML18147 and RAISE trials had received previous treatment with bevacizumab, as compared with only 30 % of patients in the VELOUR trial. The anti-VEGF agents used in these trials also differ with respect to their mechanism of action and pharmacokinetic properties. For example, bevacizumab targets VEGF-A to cause ligand sequestering; ziv-aflibercept blocks VEGF-A, VEGF-B, and PlGF using the IgG1 Fc-VEGF receptor construct; and ramucirumab targets VEGFR-2 to prevent receptor activation by VEGF-A. Despite these differences, data from these three trials provide confirmatory evidence that inhibition of tumor angiogenesis beyond initial disease progression is an effective treatment strategy in mCRC. However, questions remain: potential predictive markers for these VEGF-A/VEGFR-2 pathway inhibitors, whether colorectal cancer may develop crossover tolerability/resistance to these treat different agents, potential opportunities to the possible mechanisms of moderate benefits (~1.4 months survival advantage) of these anti-angiogenic agents in mCRC, and possibilities of these anti-angiogenic agents in combination with other biologic target oriented agents, in particular immune checkpoint inhibitors.

The efficacy of bevacizumab and aflibercept may be affected by the local concentration of targeted ligand(s). Expression of VEGF-A and other ligands may increase after the loop blockage as a physiological response to rebalance the pathway, e.g., aflibercept benefits less in those patients previously exposed to bevacizumab (Velour data). Biologic efficacy of ramucirumab may be less influenced by the local concentration of VEGF-A because of binding in the VEGFR-2 extracellular domain. A limited benefit of this class of anti-angiogenic agents may be partly due to the fact that no selection criteria have been used to enrich for those patients who may be likely to derive a durable benefit from treatment. Based on the heterogeneous nature of colorectal cancer, to identify predictive biomarkers for VEGF signaling inhibitors may potentially broaden the clinical impact of these agents and might even distinguish the clinic application of each agent. Many biomarker studies have been developed and others are ongoing; however, there has been no prospective validation reported. For example, low VEGF(165)b:VEGF(total) ratio may be a predictive marker for bevacizumab in metastatic colorectal cancer [7], and individuals with high relative levels may not benefit from bevacizumab; low serum angiopoietin-2 (Ang-2), an inhibitory ligand of endothelial Tie-2 receptor, may be a predictive marker; and low serum neuropilin-1 (NRP-1) may predict better response and overall outcome of tivozanib, an oral VEGF TKI, or bevacizumab in combination with mFOLFOX6 [8]. A meta-analysis of individual patient data from six randomized phase III trials to explore the potential relationships between 195 common genetic variants in the vascular endothelial growth factor (VEGF) pathway and bevacizumab treatment outcome suggests that variants in VEGF-A, EPAS1, IL8RA, VHL, and VEGFC have potential value in predicting bevacizumab treatment outcome [9].

So far, no selection criteria have been used to enrich for patients that might be more likely to derive durable responses from a particular anti-VEGF therapy. Consequently, optimal utilization of the available anti-angiogenesis options remains an unfulfilled goal at this time. In current clinical practice, decisions regarding the choice of agent are usually guided by physicians’ familiarity with the drug and the cost of therapy. Identification of robust clinical and molecular biomarkers to predict sensitivity, resistance, or toxicity from VEGF signaling inhibitors would be considered a significant breakthrough, as it might help distinguish the clinical application of each agent [10]. Translational research efforts targeted towards this goal are actively ongoing. Other clinically relevant questions that currently remain unanswered include identification of optimal sequencing strategy and the cost-to-benefit ratio of these anti-VEGF therapies in the management of mCRC.
